# Technique for repair of recurrent aortic valve dehiscence

**DOI:** 10.1093/jscr/rjaf077

**Published:** 2025-02-25

**Authors:** Hung Dung Van, Chi Linh Chau, Minh Chau Van Nguyen

**Affiliations:** Cardiac Surgery Department, Ho Chi Minh Heart Institute, 4 Duong Quang Trung St., Ward 12, District 10, Ho Chi Minh City 70 000, Viet Nam; Thoracic and Cardiovascular Surgery Department, University of Medicine Pham Ngoc Thach, 2 Duong Quang Trung St. Ward 12, District 10, Ho Chi Minh City 70 000, Viet Nam; Cardiac Surgery Department, Ho Chi Minh Heart Institute, 4 Duong Quang Trung St., Ward 12, District 10, Ho Chi Minh City 70 000, Viet Nam

**Keywords:** aortic valve dehiscence, infective endocarditis, atrioventricular block, Behcet's disease

## Abstract

Re-operation for aortic paravalvular leak or multiple dehiscences due to Behcet's disease or unknown causes remains a challenge. We describe a modified transmural sub-coronary suturing technique to address this issue. Between August 2022 and May 2024, the modified transmural sub-coronary suturing technique was applied in five cases of severe aortic paravalvular leak. The median follow-up period was 14 months (ranging from 8 to 19 months) with promising outcomes: no mortality, no significant paravalvular leak, no atrioventricular block, and no need of re-operation. Our transmural sub-coronary suturing technique with external Dacron strip reinforcement demonstrates good short-term outcomes. Further long-term follow-up is needed to evaluate the long-term effectiveness of this technique.

## Introduction

Aortic valve dehiscence after valve replacement has various causes, including infective endocarditis, Behcet's disease, ankylosing spondylitis, non-infectious aortitis, associated vasculitis, or unknown origins [1, 2]. Paravalvular leak often occurs early, and dehiscence tends to occur multiple times. Reoperation in these cases is consistently challenging, and multiple techniques have been employed to address it. We report the outcomes of five cases utilizing a modified technique in repeated aortic valve dehiscence of unknown causes.

### Brief technical description

**Figure 1:**
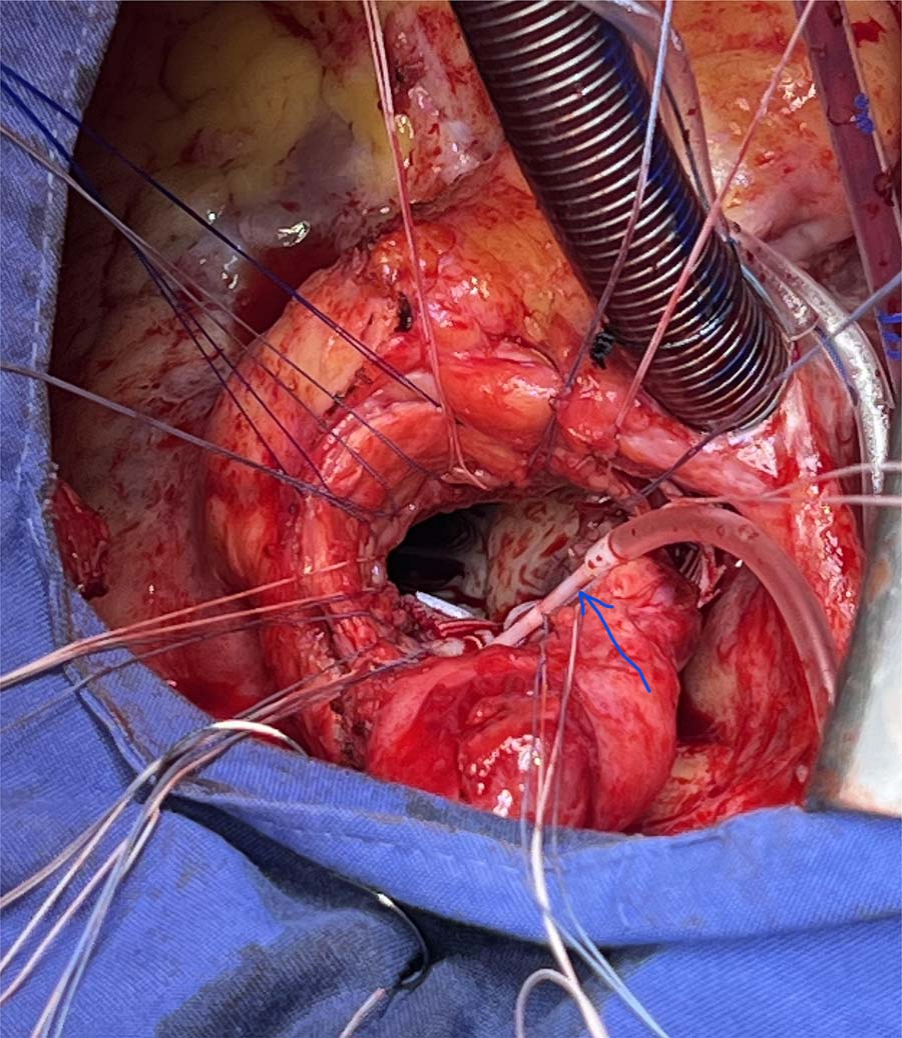
Insert a coronary cannula (arrow) into the left common coronary artery.

**Figure 2:**
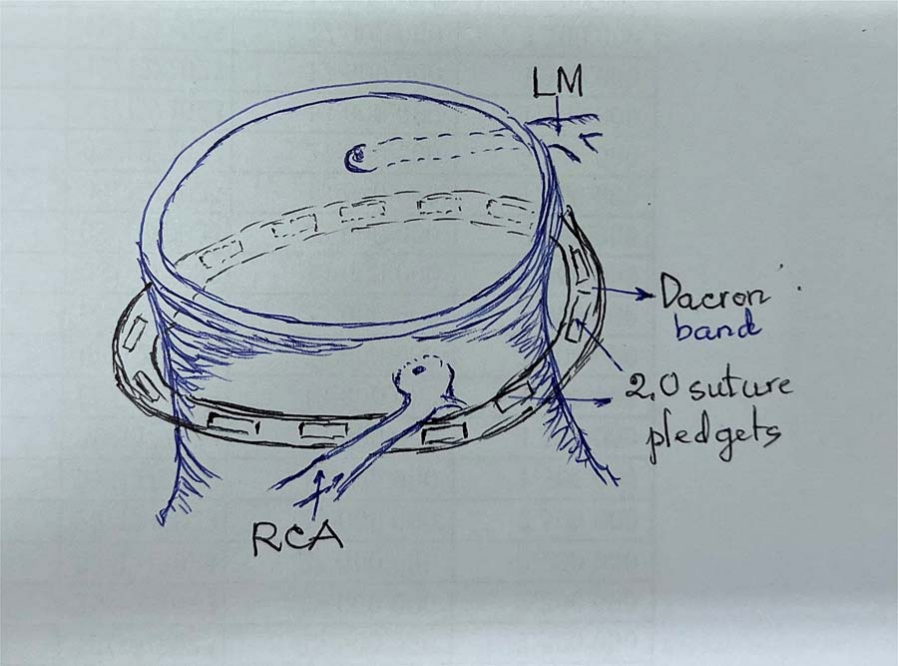
Schematic illustration of enhanced Dacron band location.

**Figure 3:**
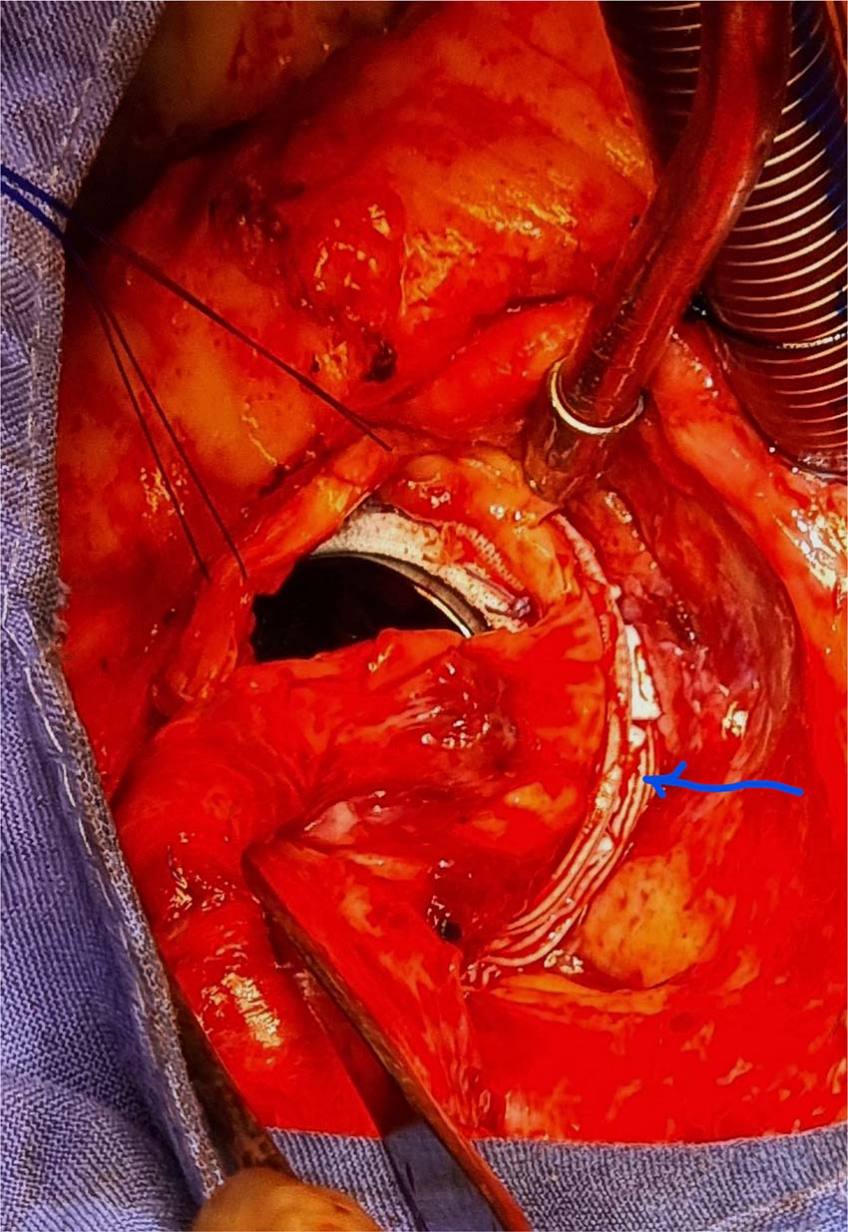
Dacron band outside after tying the suture (arrow).

- Establish extracorporeal circulation and administer myocardial protection solution (HTK Custodiol) directly into both coronary ostia.
- Dissect around the aortic root ~5–8 mm deeper than two coronary ostia. To avoid damage to the coronary arteries, using coronary cannula infusion of cardioplegia (DLP Coronary Artery Ostial Canulae, Medtronic Minneapolis USA) with size 4F to insert into each coronary artery, thus being able to orient the coronary artery correctly (Fig. 1)
- Use two Dacron strips (5–6 mm in height) to encircle the external circumference of the aorta and place them beneath the coronary ostia.
- Perform U-stitch with 2.O pledgets sutures (Ti-Cron®, Covidien with 26 mm needles) through the Dacron strips and aortic wall along the circumference of the annulus (pay attention not to cause compression of the coronary ostium, then secure the stitches to the prosthetic sewing ring (Figs. 2 and 3). Recheck the access to the two coronary ostia with a coronary cannula and close the aorta two-layer as usual.

## Results

**Figure 4:**
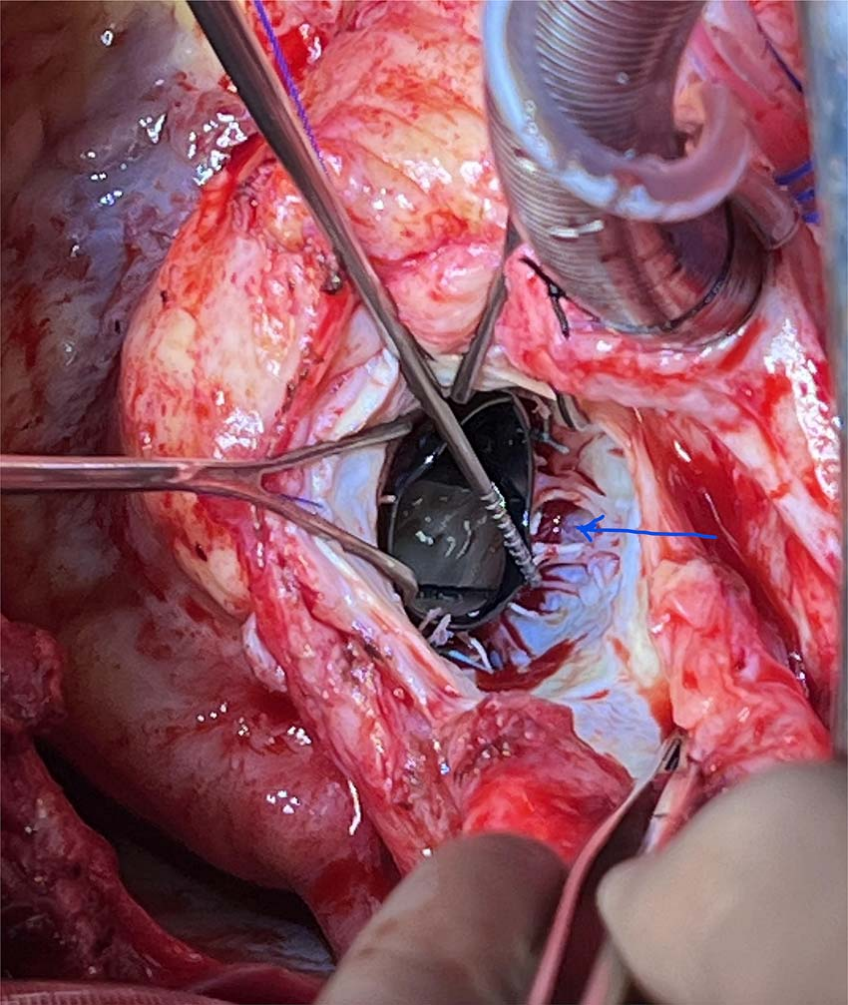
‘Spoke’ pattern dehiscence (arrow).

From August 2022 to May 2023, five cases of multiple aortic valve dehiscence were applied to this technique. The common characteristic of these cases is the absence of clinical and para-clinical evidence of infective endocarditis. The artificial valve almost did not attach to the aortic annulus during surgery. Two types of valve dehiscence were observed: (i) nearly complete dehiscence and (ii) “spoke” pattern (Fig. 4) with many points of dehiscence. All patients survived and were discharged from the hospital without atrio-ventricular block. All patients received postoperative immunosuppressive therapy with methylprednisolone (Medrol®) at 1 mg/kg for 2 weeks and decreased to 4 mg/day and maintained at this dose. The echocardiography at 1, 3, and last month showed either mild or no paravalvular leakage, and no re-operation was required. The median follow-up time was 14 months. Some characteristics of the study patient group are presented in Table 1.

**Table 1:** Characteristics of five patients in this study

Patients	Previous surgery	Evidence of Behçet	Surgical finding	CPB/ Ao clamp time (mn)	Duration from previous surgery	Echocardiography post-op (last follow up)	Immuno-suppression drugs post-op
No 1, male, 34 y.o., 55 kg	Two times of mechanical AVR	Yes	Dehiscence 1/2 aortic perimeter without vegetation	97/73	3 and 5 months	Trivial paravalvular leak	Medrol® 1 mg/kg × 2 weeks then 4 mg/day
No 2, female, 33 y.o., 56 kg	Two times of mechanical AVR	Yes	Dehiscence 1/2 aortic perimeter	115/84	5 and 8 months	No paravalvular leak	Medrol® 1 mg/kg × 2 weeks then 4 mg/day
No 3, male, 48 y.o., 65 kg	Double valve replace	No	Dehiscence 1/3 aortic perimeter without vegetation, mild mitral paravalvular leakage	110/86	10 months	No aortic and mitral paravalvular leak	Medrol® 1 mg/kg × 2 weeks then 4 mg/day
No 4, male, 55 y.o., 77 kg	Two times of mechanical AVR	Unclear	Dehiscence 1/3 aortic perimeter as ‘spoke’ like without vegetation	114/82	6 and 10 months	No paravalvular leak	Medrol® 1 mg/kg × 2 weeks then 4 mg/day
No 5, female, 35 y.o., 58 kg	Three times of mechanical AVR	Yes	Dehiscence 1/3 aortic perimeter as ‘spoke’ like	125/90	3, 6 and 10 months	Mild paravalvular leak	Medrol® 1 mg/kg × 2 weeks then 4 mg/day

## Discussion

In Behcet's disease or non-infectious aortitis, multiple surgeries often lead to destruction of the annular structure. Techniques like the Danielson or modified Bentall have been applied, but they are challenging in practice. The common character of our cases is that the aortic wall is quite thick, and the endothelium is severely damaged, so we think that the aortic wall can be used as a substitute for the aortic annulus. Our technique utilizes the aortic wall as a replacement for the annulus, reinforced with a Dacron strip for better structural support. The disadvantage of this method is that it requires a lot of dissection and cannot suture the area just below the coronary ostia because it compromises the ostia coronary. Thus, the suture inside may result in mild leakage beneath the coronary ostia. (patients number 1 and 5 in our series). We always add one or two small endocardial U-stitches below the coronary ostia to reduce the leakage. The advantage of our technique is having a shorter time of aortic clamp when compared with the Danielson or Bentall modified technique. Azuma described the technique of ‘sub annular ring reinforcement technique’ to refix the new aortic valve. In this technique of Azuma, the author created a new ring below the aortic annulus with a Dacron ring. The author applied for 3/5 Behçet’s patients and no leakage was noted after a mean of 3 years of follow-up [3]. Azuma's technique was successfully applied by Mani to a case of aortic valve dehiscence four times [4]. Jung *et al* reported seven cases using ‘the sub annular endomyocardial implantation’ technique with an average follow-up time of 7.8 years and no cases of reoperation [5]. We have tried to use this technique for one patient but 4 months after the second operation, there was severe paravalvular leakage. An alternate complex technique as Xuan *et al.* have applied the ‘Commando’ technique for two cases of Behçet’s in which, both aortic and mitral valves were replaced at the first operation [6]. In our opinion, no matter what technique is used, the ultimate goal is that the artificial valve is firmly attached without reoperation. At last, patients also need long-term steroid and other immunosuppressant treatment after surgery as in treatment for Behçet’s disease.

## Conclusion

Recurrent aortic valve dehiscence poses a significant challenge in cardiac surgery. The transmural aortic suturing technique with Dacron reinforcement has demonstrated favorable short-term outcomes. Continued follow-up is necessary to assess long-term efficacy.

## References

[1] Koo HJ, Yang DH, Kang JW, et al. Demonstration of prosthetic aortic valve dehiscence in a patient with noninfectious aortitis by multimodality imaging findings of echocardiography and computed tomography. Circulation 2013;128:759–61. 10.1161/CIRCULATIONAHA.112.000749. 23940391

[2] Pamulapati H, Janga P, Taduru S, et al. Mechanical aortic valve dehiscence and aortic root aneurysm: an interesting case with a timely diagnosis and intervention. Cureus 16:e63432. 10.7759/cureus.63432. 39077287 PMC11286104

[3] Azuma T, Yamazaki K, Saito S, et al. Aortic valve replacement in Behcet’s disease: surgical modification to prevent valve detachment. Euro J Cardiothorac Surg 2009;36:771–2. 10.1016/j.ejcts.2009.05.031. 19699654

[4] Mani GK, Kanwar JR, Sharma PK, et al. Fourth recurrence of aortic annular dehiscence following AVR for aortic regurgitation. Apollo Med 2011;8:238–42. 10.1177/0976001620110314.

[5] Jung Y, Ahn BH, Lee KS, et al. A novel solution to prosthetic valve dehiscence after aortic valve surgery in Behcet’s disease. Interact Cardiovasc Thorac Surg 2017;24:342–7. 10.1093/icvts/ivw361. 28011741

[6] Xuan J, Jinduo L, Fareed K, et al. Aortic and mitral valve surgery for infective endocarditis with reconstruction of the intervalvular fibrous body: an analysis of clinical outcomes. J Thorac Dis 2020;12:1427–36. 10.21037/jtd.2020.03.04. 32395280 PMC7212136

